# Management of a Patient with Cardiovascular Disease Should Include Assessment of Primary and Secondary Immunodeficiencies: Part 2—Secondary Immunodeficiencies

**DOI:** 10.3390/healthcare12191977

**Published:** 2024-10-04

**Authors:** Katarzyna Napiórkowska-Baran, Agata Doligalska, Magdalena Drozd, Marta Czarnowska, Dariusz Łaszczych, Marcin Dolina, Bartłomiej Szymczak, Oskar Schmidt, Zbigniew Bartuzi

**Affiliations:** 1 Department of Allergology, Clinical Immunology and Internal Diseases, Collegium Medicum Bydgoszcz, Nicolaus Copernicus University Torun, 85-067 Bydgoszcz, Poland; zbartuzi@cm.umk.pl; 2 Student Research Club of Clinical Immunology, Department of Allergology, Clinical Immunology and Internal Diseases, Collegium Medicum Bydgoszcz, Nicolaus Copernicus University Torun, 85-067 Bydgoszcz, Poland; doli285@wp.pl (A.D.); magdalena.drozd515@wp.pl (M.D.); czarnowska.marta@onet.pl (M.C.); laszczychdariusz@gmail.com (D.Ł.); melminio@wp.pl (M.D.); bartlomiej.szymczak1@gmail.com (B.S.); okischmidt@wp.pl (O.S.)

**Keywords:** secondary immunodeficiencies, immune defects, cardiovascular diseases, heart diseases

## Abstract

Background: Cardiovascular diseases are among the most common chronic diseases, generating high social and economic costs. Secondary immunodeficiencies occur more often than primary ones and may result from the co-occurrence of specific diseases, treatment, nutrient deficiencies and non-nutritive bio-active compounds that result from the industrial nutrient practices. Objectives: The aim of this article is to present selected secondary immunodeficiencies and their impact on the cardiovascular system. Results: The treatment of a patient with cardiovascular disease should include an assess-ment for immunodeficiencies, because the immune and cardiovascular systems are closely linked. Conclusions: Immune system dysfunctions can significantly affect the course of cardiovascular diseases and their treatment. For this reason, comprehensive care for a patient with cardiovascular disease requires taking into account potential immunodeficiencies, which can have a significant impact on the patient’s health.

## 1. Introduction

Secondary immunodeficiencies (SIDs) are more common than inborn errors of immunity (IEI) and may result from the presence of certain chronic diseases, nutrient deficiencies, and non-nutritional bioactive compounds that result from industrial dietary practices and medical treatments. It is becoming increasingly clear that immunodeficiencies may be responsible for certain cardiovascular diseases, their accelerated progression, and the difficulty in their control. Early detection of deficiencies not only improves the quality and longevity of patients, but also allows for better control of cardiovascular diseases and even their prevention.

Secondary immunodeficiencies are either temporary or permanent immune disorders. Their causes can be traced to various conditions that disrupt the body’s homeostasis. Although the cause of some SIDs can be removed, patients may still experience complications such as cardiovascular disease, among others, for the remainder of their lives. When diagnosing cardiovascular disease, it is essential to determine whether the disorder is due to the cause of the SID or whether immune dysfunction is directly at the direct cause.

The causes of secondary immunodeficiencies are presented in Table 1.

## 2. Selected Chronic Diseases Associated with Increased Cardiovascular Risk

The following section provides an overview of selected chronic diseases associated with the occurrence of secondary immunodeficiency that may affect cardiovascular risk. A detailed discussion of all diseases is beyond the scope of this review. Therefore, the most common diseases and/or those that constitute a significant clinical problem were selected.

### 2.1. Type 2 Diabetes Mellitus (T2DM)

Type 2 diabetes mellitus (T2DM) is a metabolic disorder that causes abnormal glycemia. Increased blood glucose concentration, resulting from improper secretion or action of insulin, leads to numerous disorders of protein and fat metabolism. In the pathogenesis of type 1 diabetes (T1DM), insulin production is inhibited due to the destruction of pancreatic β cells during the autoimmune process [7]. T2DM predisposes to diabetic cardiomyopathy and atherosclerotic cardiovascular disease (CVD), which increases the likelihood of developing heart failure and myocardial infarction [8]. Both T1DM and T2DM significantly affect the immune system and lead to dysfunctional immune responses [9,10].

It has been proven that monocytes ”sola’ed from individuals with T1DM and T2DM after stimulation with lipopolysaccharides (LPS) secreted less interleukin 1 beta (IL-1β) compared to the control group. Moreover, monocytes isolated from T1DM patients secreted lower levels of interleukin 1 (IL-1) and interleukin 6 (IL-6) compared to healthy donors [11,12]. However, the findings of the study unveiled that individuals diagnosed with T2DM exhibited increased levels of IL-6, and tumor necrosis factor alpha (TNF-α) in comparison to those without diabetes [13]. Additionally, alterations in the concentrations of other immunological markers were noted, including decreased interleukin 7 (IL-7) concentrations. The concentrations of IL-12, IL-15, macrophage-derived chemokine (MDC), and C-reactive protein (CRP) increased with body weight. Moreover, there was a positive correlation between TNF-α and HbA1c levels [14]. The level of interleukin 10 (Il-10) is also significantly reduced in patients with T2DM [15]. Interleukin 6 assumes a pivotal role in safeguarding against pathogens and orchestrating the adaptive immune response by stimulating antibody generation and the development of effector T-cells [16]. It stands as a central mediator of the acute phase reaction and serves as a principal driver of hepatic synthesis of C-reactive protein. Heightened concentrations of IL-6 are correlated with escalated risks of future myocardial infarction (MI), a risk that escalates proportionally with the elevation of this interleukin [17]. Furthermore, it has been demonstrated that elevated levels of CRP and IL-6 serve as autonomous prognosticators of forthcoming cardiovascular risk, particularly coronary heart disease (CHD) [18]. Another investigation substantiated that heightened IL-6 levels are linked to worse prognosis in unstable angina and post-acute myocardial infarction scenarios. Elevated IL-6 levels are strongly correlated with future cardiac events and mortality in populations with stable coronary artery disease (CAD) during long-term observation [19].

Tumor necrosis factor (TNF), a pivotal player in cardiovascular pathology, exerts a significant influence [20]. Elevated levels of TNF-α have been observed in various cardiovascular conditions, such as heart failure, myocarditis, coronary heart disease, and myocardial infarction [21,22]. TNF-α instigates myocardial damage through multifaceted mechanisms, encompassing the promotion of cardiomyocyte apoptosis, acute necrosis, impairment of contractile function, and stimulation of fibrosis [23,24]. Its activation further contributes to hypertrophy, and ultimately leads to ventricular remodeling [25]. This, in turn, exacerbates cardiac dysfunction and advances the progression of heart failure [26,27]. Moreover, TNF-α has been linked to the development of arrhythmias. Its involvement in atrial remodeling further highlights its potential role in the pathogenesis of atrial fibrillation [28], where it perturbs the cardiac electrical properties, disrupting conduction and rhythm [29].

Interleukin 10 is an anti-inflammatory cytokine that plays a crucial role in regulating the immune response and inflammation. When present in lower concentrations, particularly in the context of cardiovascular diseases, several important implications arise [30,31]. IL-10 acts as a natural suppressor of inflammation by inhibiting the production of pro-inflammatory cytokines such as TNF-α, IL-1, IL-6, and interleukin-8 (IL-8) [32]. Lower concentrations of IL-10 may impair the resolution of inflammation in vascular tissues, leading to prolonged and chronic inflammatory responses that contribute to the enhanced atherosclerosis [33] and the progression of cardiovascular diseases [34]. Studies have suggested that lower levels of IL-10 are associated with an increased risk of cardiovascular events, such as myocardial infarction, heart failure, and stroke [35,36,37,38]. This association underscores the importance of IL-10 in maintaining cardiovascular health and preventing adverse outcomes. The conducted study on mice, aimed at assessing the administration of exogenous IL-10 as a therapeutic intervention for diabetic myocardial infarction, demonstrated compelling results. It revealed that IL-10 substantially diminished infarct size and ameliorated cardiac function in diabetic mice. Additionally, IL-10 fostered an enhancement in capillary density, a reduction in apoptosis, and a mitigation of inflammation within the border zone of the infarcted hearts. Understanding the role of IL-10 in regulating immune responses and inflammation is essential for developing therapeutic strategies aimed at mitigating the progression of cardiovascular diseases and improving patient outcomes [39].

Diabetes mellitus affects neutrophils, significantly disturbing their functioning and the role they serve in immunological processes. Increased serum resistin levels in T2DM patients lead to decreased reactive oxygen species production by neutrophils [40,41]. Hyperglycemia may affect neutrophil functionality. Decreased neutrophil degranulation has been reported with elevated glucose concentrations [42]. Furthermore, the neutrophil function of producing neutrophil extracellular traps (NETs) and phagocytosis was inhibited under hyperglycemic conditions. There are reports indicating that hyperglycemia can increase the formation of NETs. Studies suggest that under hyperglycemic conditions, especially in type 2 diabetes, there can be increased activation of neutrophils, leading to excessive NET formation. This mechanism is linked to chronic oxidative stress and inflammation that accompanies hyperglycemia. High glucose levels lead to the production of reactive oxygen species (ROS), which are important inducers of NET formation. In patients with diabetes, higher NET concentrations have been shown to contribute to complications such as atherosclerosis and other vascular diseases. In summary, in some cases, hyperglycemia can both inhibit the defensive functions of neutrophils, such as phagocytosis, and increase NET formation, which may contribute to pathological inflammatory conditions. [43,44]. A decrease in the effectiveness of immunoglobulin-mediated opsonization and neutrophil migration was also observed [45,46]. Neutrophils facilitate tissue repair through the clearance of cellular debris and potential pathogens, promoting angiogenesis, and directing macrophages toward the regenerative M2 phenotypes [47,48]. As evidenced by a study, their pivotal role in cardiac repair is underscored by observations of deteriorated cardiac function, heightened fibrosis, and the onset of heart failure in neutrophil-depleted mice following myocardial infarction. Consequently, any disruption in their function could substantially impact the risk of cardiovascular disease [49].

### 2.2. Obesity

Nowadays, obesity poses a formidable challenge, presenting a significant hurdle. It substantially elevates the risk of chronic diseases and cardiovascular diseases. Recent research underscores the profound effects of obesity on immunity and defense against pathogens, such as disrupting lymphoid tissue integrity, modifying leukocyte development and activity, and affecting the coordination between innate and adaptive immune responses [50,51]. Obesity-induced chronic low-grade inflammation contributes to the development and progression of cardiovascular disease. Inflammatory cytokines released by adipose tissue, such as IL-6 and TNF-alpha, promote endothelial dysfunction, atherosclerosis, and plaque instability, all of which are key factors in the development of cardiovascular disease [52]. 

Studies indicate that obesity diminishes the variety of circulating T cells, thereby restricting their ability to respond to a wide range of pathogenic antigens [53,54]. Moreover, obesity has been found to decrease the size of inguinal lymph nodes, impede the transport of lymphatic fluid, hinder dendritic cell migration to peripheral lymph nodes, and lower the T cell count in lymph nodes [55]. Leptin resistance accompanies obesity [56], impacting the regulation of bone marrow hematopoiesis [57]. This deficiency is linked to reduced hematopoiesis, diminished T cell production, and compromised immunity [58,59]. Leptin has demonstrated dual effects on T lymphocyte production and development in the thymus, as well as the identification of T lymphocyte subsets in lymph nodes [60]. Hence, obesity disrupts immune system integrity and induces alterations in leukocyte development, migration, and diversity.

Interleukin-1β (IL-1β) is a pro-inflammatory cytokine belonging to the IL-1 interleukin family and is upregulated in obesity [61]. IL-1β is synthesized by adipocytes, macrophages, and neutrophils, and plays a prominent role in conditions such as atherosclerosis, acute myocardial infarction, myocarditis, and chronic or decompensated heart failure [62,63]. Cytokines from the IL-1 family exert influence on the systolic function of the heart ventricles. IL-1β has been demonstrated to reduce the responsiveness of beta-adrenergic receptors on L-type calcium channels through a mechanism independent of cyclic adenosine monophosphate (cAMP) [64]. Furthermore, IL-1β has been observed to reversibly decrease the expression of genes crucial for calcium regulation in cardiac muscle [65]. Studies conducted on animal models have revealed reversible systolic dysfunction and reduced contractile reserve in the left ventricle following single or multiple injections of IL-1β in initially healthy mice. The results of this experiment elucidate the cardiodepressive impact of IL-1β [66]. In addition, in cases of fulminant myocarditis, the release of IL-1β triggers extensive inflammation throughout the myocardium. This inflammation contributes to the additional demise of cardiomyocytes, gradual deterioration of viable contractile tissue, and the onset of cardiomyopathy and subsequent heart failure [67].

### 2.3. Rheumatoid Arthritis

Cardiovascular disease (CVD) is the leading cause of mortality in patients with rheumatoid arthritis (RA). Aviña-Zubieta et al. observed that the mortality rate for CVD in this group of patients was 50% higher than in the general population [68,69]. Furthermore, gender has an additional negative impact on prognosis. It has been demonstrated that female gender is a factor in the increased frequency and severity of RA [70].

In autoimmune diseases, where there is a chronic inflammatory process caused by an imbalance in the formation of pro-inflammatory and anti-inflammatory molecules, endothelial activation occurs, thereby increasing the rate of atherosclerotic lesions. In progressive rheumatoid arthritis, there is an increase in pro-inflammatory cytokines such as IL- 1β, IL-6, tumor necrosis factor (TNF)-α, and interferon (IFN)-γ, which are most likely derived from the synovial membrane. This is followed by the binding of monocytes, neutrophils, and platelets to the activated vascular endothelium [71,72,73].

Interferon-gamma (IFN-γ) is a cardinal molecule involved in the rupture of atherosclerotic plaques. In the event of an increase in this pro-inflammatory cytokine, the T lymphocytes within the plaque reduce the production of collagen by the cells that constitute the muscular layer of the vessel, thereby thinning the blood vessel. The CD40L molecule produced by activated T cells induces foam cells to produce two enzymes. These collagenases and gelatinases degrade the extracellular matrix. Upon rupture, a thrombotic plug is formed, resulting in obstruction and the subsequent development of acute cardiovascular conditions [74,75]. 

Additionally, matrix metalloproteinases (MMPs) also play a significant role in degrading the extracellular matrix [76]. Collagen is a key structural component of fibrous cap, which covers the atheroma. The more collagen there is in the cap, the thicker it becomes and the probability of it rupturing decreases [77]. MMPs, especially MMP-2 and MMP-9, which belong to gelatinases, cause degradation of collagen leading to thinning of the protective cap [76,78]. Plaque vulnerability is increased and the risk of rupture becomes high. Finally, the erosion of the fibrous cap by MMPs exposes the plaque’s core to the bloodstream, further promoting clot formation, by releasing tissue factor and contributing to acute cardiovascular events [74].

In addition to the conventional cardiovascular disease (CVD) risk factors observed in rheumatoid arthritis (RA) patients, particular attention should be directed towards the presence of extra-articular symptoms, erosions, and a prolonged disease duration. Consequently, the early implementation of preventive measures and the treatment of RA patients is of paramount importance [76,77,78]. The conventional cardiovascular risk factors do not fully explain the phenomenon of early CVD development in RA. Habets et al. observed an increased incidence of active platelets in RA [79]. In the context of the disease, activated platelets exhibit inflammatory and hemostatic functions. These particles act as an activator for Inflammatory adhesion receptors, cytokines, and matrix metalloproteinases [80]. Additionally, activated platelets secrete proteins that cause their reciprocal aggregation to form a fibrin clot [81].

Tissue factor (TF) is a glycoprotein that exhibits increased expression in the syno-vial membrane of patients with rheumatoid arthritis (RA). In addition, it is found in pro-inflammatory mediators such as B lymphocytes and macrophages [81,82,83]. The aforementioned protein also has elevated expression due to its procoagulatory effect on vascular endothelium in response to the action of pro-inflammatory cytokines [84]. In response to constant contact with pro-inflammatory cytokines, fibrin thrombi formed in RA exhibit protrusions on their surface. These protrusions result in significantly decreased fibrin stability and increased fibrin permeability which may lead to a more persistent and unstable thrombotic environment, favoring the development of CVD [85].

In patients with rheumatoid arthritis (RA), two processes occur that reinforce each other. Factors that influence the formation of thrombi, such as fibrinogen and plasminogen activator inhibitors, are elevated which contribute to a disrupted balance between coagulation and fibrinolysis. This disruption can lead to chronic thrombus formation and hinder the ability to effectively resolve clots, thereby increasing the risk of CVD [86]. Furthermore, chronic inflammation activates the thrombin-activatable fibrinolysis inhibitor (TAFI), thereby maintaining the formed fibrin. Additionally, the interference of citrulline–fibrinogen complexes with plasmin activity further exacerbates this risk of developing CVD by impeding the fibrinolytic process [87,88,89,90]. In the context of RA, these changes in thrombus characteristics not only exacerbate local inflammation but also pose a risk for systemic thromboembolic events. Therefore, understanding these interactions is crucial for developing targeted therapeutic strategies to mitigate cardiovascular complications in RA patients.

### 2.4. Systemic Lupus Erythematosus

Another disease entity that affects the cardiovascular system is systemic lupus erythematosus (SLE), which, along with rheumatoid arthritis, is a cause of hypertension and other cardiovascular diseases [91]. Han et al. observed the existence of hypertension in 31% of people with RA compared to 23% in the general population [92]. In the case of SLE, hypertension is observed in 40% of patients under the age of 40 [91]. SLE patients often face vitamin D3 deficiency due to avoidance of sunlight, which is related to skin hypersensitivity. Sabio et al. demonstrated that vitamin D3 deficiency can affect the development of a non-dipper pattern in SLE patients [93].

Metabolic diseases such as insulin resistance and hypercholesterolemia frequently coexist with SLE. In these disease entities, elevated levels of adipokines are observed, one of which is leptin. Elevated leptin levels are reflected in disease activity and reduced levels of regulatory T lymphocytes. Low levels of the aforementioned lymphocytes have been demonstrated to have a proven link to the development of hypertension [94].

Circulating autoantibodies and pro-inflammatory mediators affect the adhesion of cells to the surface of blood vessels. Endothelial cell adhesion molecule-1 (VCAM-1) and intracellular adhesion molecule-1 (ICAM-1) are transmembrane proteins that trigger the migration of immune cells to the vessel walls. Activated by immune complexes, platelets further contribute to the development of inflammatory reactions by releasing mediators. Collectively, these processes create a proatherogenic environment on the epithelial surface [95].

Antiphospholipid antibodies and their immune complexes, formed in SLE, act as prothrombotic factors. Platelets are the site of binding complexes with phospholipids and β2-glycoprotein, among others. This results in the release of granules from platelets and, consequently, procoagulant complexes. Acute proteins such as fibrinogen maintain inflammation, which is one of the factors in thrombosis. Le Minh et al. showed that anti-dsDNA antibodies cause activation and cross-linking due to fibrinogen and contraction of the clot consisting of platelets [96].

### 2.5. Inflammatory Bowel Disease

Inflammatory bowel disease (IBD) is an autoimmune disorder that affects the cardiovascular system. Mechanisms that influence complications beyond the gastrointestinal tract include chronic inflammation with oxidative stress, impaired platelet and vascular endothelial function, and intestinal dysbiosis, among others [97,98,99].

Activation of pro-inflammatory T helper lymphocyte pathways (Th-17 and Th-1) enhances the production of inflammatory cytokines. Increased expression of toll-like receptors -2 and -4 further augment the production of interleukins (especially IL-6 and IL-12). The consequence of the aforementioned process is vascular endothelium deterioration due to oxidative stress, which is one of the critical causes of widespread cardiovascular consequences [100]. Vascular endothelial growth factor (VEGF), also activated by inflammatory interleukins (especially by IL-1 and -6), affects the vascular endothelium by increasing the expression of adhesion molecules, which include ICAM-1, MCP-1, and E-selectin. Subsequently, the endothelium undergoes apoptosis, remodeling, and fibrosis. Exacerbations, as well as the development of IBD itself, correlate with an increase in C-reactive protein (CRP), which affects the development of atherogenesis. CRP levels above 5 mg/L are considered a predictor of potential cardiovascular events [101].

Microbial imbalances in the gut lead to inflammatory bowel disease and cardiovascular disease. Disturbances in the Firmicutes/Bacteroidetes ratio can lead to hypertension. Opportunistic bacteria increase the risk of ischemic stroke and TIA, while Enterobacteriaceae increase the general risk of cardiovascular disease [101,102,103]. Dysbiosis itself is a factor in the disruption of intestinal permeability. As a consequence, lipopolysaccharides enter the blood and increase inflammation, which is one of the main factors in the onset of atherosclerosis [104,105]. In addition, mucus secretion and fiber fermentation are impaired, which increases inflammation. Pro-inflammatory cytokines cause a pro-thrombotic state due to increased levels of thrombin [104].

Pericarditis as a disease state occurs almost exclusively as an adverse reaction to drugs used in the treatment of IBD. The mechanism of these complications is related to IgE-mediated reactions that have toxic effects on the pericardium and cardiomyocytes [105]. The persistent inflammation in the organism due to IBD affects myocardial remodeling, resulting in valvular abnormalities and further systolic dysfunction with reduced stroke volume [106].

Aniwan et al. observed that the level of gastrointestinal involvement in IBD correlates with the incidence of myocardial infarction risk. In patients with an involved colon, the risk of myocardial infarction increases three-fold [107].

Females have a higher cardiovascular risk. This is presumably related to a more significant immune response and higher levels of C-reactive protein. IBD patients have higher levels of highly sensitive CRP and fibrinogen. An additional factor is age; younger patients due to earlier onset of inflammation are burdened with greater cardiovascular consequences in long-term follow-ups [108,109].

High cholesterol is one of the leading cardiovascular risk factors; however, IBD patients have lower levels of total cholesterol and LDL cholesterol in comparison to the general population, while triglycerides and cholesterol levels are constant in HDL lipoproteins. Nevertheless, Shen et al. proved that the lipid profile of people struggling with IBD is more proatherogenic [110,111] Chen et al. observed that the overall serum lipid levels of IBD patients negatively correlated with the severity of the disease [112]. 

### 2.6. Neoplasms

Cardiovascular disease and neoplasms are intricately linked, sharing common mechanisms and risk factors that contribute to the onset and progression of both conditions. Modifiable risk factors such as hypertension, diabetes mellitus, obesity, smoking, dietary habits, physical inactivity, and social determinants of health are pivotal in predisposing individuals to both CVD and cancer. Notably, cancer patients face a heightened risk of developing various cardiovascular diseases and succumbing to CVD, largely due to the emergence of these shared risk factors. Conversely, individuals with CVD also demonstrate an increased susceptibility to different types of cancer and cancer-related mortality, though this association varies across different studies and cancer types. The underlying mechanisms connecting CVD and cancer encompass chronic inflammation [113]. 

Robust associations have been observed among the incidence of cardiovascular diseases, the onset of cancer, and the performance of the immune system. The dysregulation of immune markers seen in cardiovascular diseases markedly influences cancer pathogenesis. Consequently, IL-12 and IFNγ inhibit tumorigenesis, contrasting with the tumor-promoting effects of IL-6, IL-17, and IL-23. Similarly, TNF-α, TGF-β, and IL-6 foster the growth and survival of cancer cells [114,115].

It has been demonstrated that patients with cardiovascular disease are more likely to develop malignant tumors compared to those without cardiovascular conditions. Furthermore, atherosclerosis significantly elevates the risk of cancer. In those individuals where atherosclerosis was involved in the development of CVD, it was found that there was an increased risk of developing cancers of the lung, bladder, colon, head and neck, liver, prostate, pancreas, and kidney, as well as lymphoma, leukemia, and other hematological malignancies. However, a significantly lower risk of breast, ovarian, and uterine cancer was observed [116]. 

Furthermore, recent preclinical studies employing murine models of cardiovascular diseases such as heart failure, cardiac remodeling, or myocardial infarction have shown that solid tumors exhibit accelerated growth when cardiovascular abnormalities are present. Additionally, cardiac remodeling not only supports tumor growth but also metastasis, thereby advancing cancer progression [117,118,119,120]. 

The relationship between cardiac-specific and inflammatory biomarkers and the incidence of new-onset cancer has been investigated. Mice exhibiting higher levels of NT-proBNP demonstrated an elevated risk of developing both all-cause cancer and colorectal cancer compared to those with lower NT-proBNP levels. Moreover, the research revealed that both cardiac and inflammatory biomarkers, in both unadjusted and adjusted models, were linked to new-onset lung cancer and cancers of the male reproductive system. Cardiac biomarkers alone were found to be associated with cancers of the female reproductive system [118].

Myocardial infarction induces an epigenetic reprogramming of Ly6Chigh monocytes in the bone marrow, leading them to adopt an immunosuppressive phenotype. This phenotype is maintained transcriptionally in monocytes present in both the circulation and tumors. Additionally, MI increases the levels of circulating Ly6Chigh monocytes and their recruitment to tumors. The reduction of these cells alleviates MI-induced tumor growth, indicating that MI influences the innate immune system and contributes to the progression of breast cancer [119].

Myocardial infarction induces the production of interleukin 1β, which, along with β3 adrenergic stimulation, activates leukocyte progenitors in the bone marrow. This activation leads to a temporary increase in the number of innate immune effector cells, particularly monocytes, within the bloodstream and hematopoietic reservoirs [120]. Monocytes play a crucial role in regulating the tumor microenvironment, including facilitating tumor immune evasion, angiogenesis, as well as tumor cell proliferation, migration, invasion, and metastasis. Therefore, higher levels of circulating monocytes are associated with poor clinical outcomes in various cancers [121,122]. The administration of anti-interleukin-1ß antibody in lung cancer patients yielded decreased adverse cardiovascular outcomes. Additionally, a consequential effect was observed in the reduction of both lung cancer incidence and mortality rates [123,124]. Although targeting IL-1β shows potential in lowering cardiovascular events and occurrences of cancer, this strategy is accompanied by a slight uptick in the risk of infections [125]. In recent findings, macrophage checkpoint inhibitors, tailored to reactivate the body’s anticancer immune vigilance in lymphoma patients, have exhibited a dual benefit by also diminishing vascular inflammation in these individuals [126].

### 2.7. HIV Infection

One of the most frequently discussed deficiencies in the literature is that caused by HIV infection. Individuals infected with human immunodeficiency virus (HIV) have a higher risk of developing cardiovascular disease, a condition that has been extensively documented. Due to the introduction of new drugs, infection with this virus is no longer such a big problem, especially in highly developed countries. The inflammatory process has a prominent role in the pathogenesis of atherosclerotic conditions, including the initiation, progression, and destabilization of atherosclerotic plaques. Inflammation leads to atherosclerotic plaque rupture resulting in acute as well as chronic coronary artery disease. In the development of heart failure, cell damage, microcirculatory dysfunction, and inflammatory infiltration within the myocardium enhance the development of inflammation, which reduces systolic and diastolic function of the heart [127,128,129,130]. The advent of modern pharmaceuticals has significantly diminished the significance of viral infections, yet regrettably, novel hazards have emerged, including deficiencies in essential nutrients and non-nutritive bioactive compounds resulting from the practices of the food industry. Unfortunately, a considerable number of malnourished people are observed, despite having normal or even excessive body weight. Other significant threats include the increasing prevalence of chronic diseases and the growing number of treatments being used [131,132].

## 3. Medicaments

Secondary immunodeficiency (SID) may be caused by various agents, including immunosuppressants, used in the therapy of autoimmune disorders, neoplasms, allergic diseases, and post-transplantation to prevent graft rejection. It is worth mentioning that SID caused by used drugs is also referred to as iatrogenic (associated with medical intervention) immunodeficiency [1]. Immunosuppressants reduce inflammation and alleviate symptoms by suppressing the immune response of the host. Furthermore, immunosuppressant drugs with antiproliferative properties are used in oncology and hematology. On the other side, immunosuppressive medications are associated with numerous side effects and exacerbation of patients’ comorbidities, i.e., diabetes mellitus, hypertension, chronic kidney disease, dyslipidemia, and many more [133]. In this paragraph, we discuss the impact of selected immunosuppressants on the cardiovascular system and possible interventions that may decrease cardiovascular risk (CVR) during immunosuppressive therapy. A detailed list of drugs that may cause SID is presented in Table 1. 

### 3.1. Glucocorticoids (GCs)

Glucocorticoids (GCs) are one of the most widely used anti-inflammatory and immunosuppressive agents. Their immunosuppressive activity is associated with both the upregulation of anti-inflammatory mediators including IL-10, IL-12, an inhibitor of transcription factor NFκB (IκBα), and the downregulation of pro-inflammatory molecules IL-1, IL-2, IL-6, and tumor necrosis factor α (TNF-α) [134,135]. GCs induce alternation in the white blood cell count (WBC), including the decrease of lymphocytes (lymphopenia) and increase of immature neutrophils (neutrophilia), which cause the increase of WBC (leukocytosis) [136]. Numerous adverse events associated with the usage of GCs limit the clinical application of these agents. GCs, through a significant impact on metabolism and physiology, affect the cardiovascular (CV) system and increase the risk of cardiovascular diseases (CVD). The one-year and five-year cumulative risks of all-cause CVD are increased, respectively, to almost 9% and 28.0% when the daily prednisolone-equivalent dose is equal to or more than 25.0 mg. What is interesting, patients with low doses of GCs (less than 5.0 mg of prednisone-equivalent dose) have two times more risk of CVD [137]. Therefore, patients who are on GC therapy require particular care and clinical vigilance. Moreover, these patients require personalized CV risk prevention strategies. To evaluate the 10-year risk of CVD among patients on chronic GC therapy, it is recommended to use dedicated risk models such as the QRESEARCH Risk Estimator V.3 (QRISK3) [138]. According to recently published recommendations of the Italian Society for Rheumatology, patients with rheumatoid arthritis on prednisone dose or equivalent ≥7.5 mg/day or ≥3 months of continuous therapy should have a CVR assessment at least once a year. In addition, GCs should be used at the lowest effective dose and the shortest possible duration [139]. A recently published cohort study in England shows that chronic GCs therapy increases the risk of hypertension. During a median follow-up of 6.6 years, 34.8% of patients developed hypertension and incidence increases with a higher cumulative glucocorticoid dose. These results suggest that patients requiring chronic GC administration should be closely monitored for early detection and appropriate treatment of GC-induced hypertension. However, to determine the frequency of blood pressure measurement in such patients, more research is needed [140]. GC-induced hypertension risk is increased in particular if the daily dose of prednisolone-equivalent is >7.5 mg [141]. According to the management of GC-induced hypertension, both angiotensin-converting enzyme inhibitors and angiotensin receptor blockers are considered first-line treatments because of their cardioprotective effects. If blood pressure remains uncontrolled, the addition of calcium channel blockers or mineralocorticoid receptor antagonists should be considered [142]. GCs are also associated with an increased risk of diabetes mellitus (DM). A recently published meta-analysis of 37 studies indicates that GCs use significantly increases the risk of DM development in patients with SLE [143]. Notably, glucocorticoids are typically administered in the morning and exhibit peak activity in the evening. Consequently, glycemic measurements should be conducted as a screening test for GC-induced hyperglycemia and diabetes mellitus at 4–5 p.m. [144]. Screening for DM ought to be conducted prior to the commencement of glucocorticoid therapy and within one to three days of the initiation of treatment. Subsequent monitoring should be conducted at 3- to 6-month intervals in the first year and then annually. It is recommended that GC-induced DM be diagnosed based on the results of postprandial glycemia 2 h after a meal or an oral glucose tolerance test. The treatment strategy must be tailored to the individual patient, taking into account the specific type of glucocorticoid used, the daily dose, the treatment schedule, and the patient’s comorbidities [145]. A recently published meta-analysis indicates that an intensive glucose-lowering strategy in hospitalized patients with GC-induced hyperglycemia is not associated with an increased risk of hypoglycemic events. It seems that intermediate-acting insulin is equally effective as the basal–bolus insulin strategy. Interestingly, the addition of metformin may decrease the risk of GC-induced metabolic side effects and lower the rate of infections in hospitalized patients [146]. Glucocorticoid usage is associated with the impairment of lipid metabolism and the development of premature atherosclerosis. Chronic GC therapy is associated with an increase in total cholesterol, low-density lipoprotein cholesterol (LDL-C), and triglycerides (TG) and a decrease in high-density lipoprotein cholesterol (HDL-C). Moreover, the higher the daily GC dose, the greater the lipid metabolism disorder is observed [147]. In a recently published cross-sectional study among SLE patients, it has been shown that the duration of GC use, together with other CV risk factors such as hypertension, is an independent indicator of premature atherosclerosis development [148]. In a meta-analysis of 29 studies, it has been demonstrated that glucocorticoid treatment in patients with rheumatoid arthritis is a significant risk factor for coronary artery disease, which is one of the most common manifestations of atherosclerosis [149]. 

### 3.2. Methotrexate

Methotrexate (MTX) is an antiproliferative and immunosuppressive drug used in rheumatoid arthritis (RA), juvenile idiopathic arthritis, multiple sclerosis, psoriasis, organ transplantation, oncology, and hematology. The antiproliferative activity of MTX is associated with the inhibition of folate-dependent enzymes involved in methionine, folate, and nucleotide synthesis biochemical pathways. However, the mechanisms underlying its anti-inflammatory and immunosuppressive therapeutic effect still need to be fully elucidated [150]. MTX is used as a first-line treatment for RA in low-dose regimens. Low-dose MTX treatment is usually well-tolerated and has high clinical efficacy with a minor risk of adverse events affecting the gastrointestinal system, liver, and blood marrow function [151]. There is growing evidence of its beneficial influence on the cardiovascular system. A recently published meta-analysis indicates that MTX can reduce the mortality associated with CVD in patients with RA. This beneficial effect on the cardiovascular system may be linked with the anti-inflammatory activity of MTX, as CVD development is associated with ongoing inflammation including increased concentration of pro-inflammatory cytokines and C-reactive protein and activation of various immune cells [152]. In a meta-analysis of 10 studies, Sun et al. found that MTX treatment can prevent cardiovascular events (CVE) in RA patients. Furthermore, MTX due to its anti-inflammatory activity may be a potential agent used in the prevention of CVE in patients with coronary heart disease. However, additional research is required to determine the role of MTX in this indication [153]. MTX is also associated with a decreased risk of hypertension in patients with RA [154]. In addition, according to a recent meta-analysis, MTX treatment reduces the risk of DM2 in RA patients. The potential anti-diabetic effect of MTX is associated with, e.g., an increase in insulin sensitivity and a decrease in both hepatic gluconeogenesis and intestinal glucose absorption [155]. In a recent preclinical study in the murine model, it has been demonstrated that MTX decreases the concentration of inflammation markers, including high-sensitivity reactive protein C (hs-PCR), IL-1, IL-6, TNF-α. Furthermore, MTX treatment was associated with a significant decrease in TC, LDL-C, and TG and an increase in HDL-C. Taken together, MTX reduces cardiovascular risk by decreasing inflammation and promoting a beneficial lipid profile [156]. MTX usage has also been proven to decrease the risk of atherosclerosis development. It has been demonstrated that MTX used in doses ≥20 mg/week significantly decreases both carotid and femoral intima–media thickness [157]. Chronic inflammatory diseases such as RA favor the development of cardiac arrhythmias, which are independent cardiovascular risk factors. The most frequent arrhythmia in RA patients is atrial fibrillation (AF). The incidence of AF in patients with RA is approximately 40% higher compared to the general population [158]. Recently, Kim et al. showed that the use of MTX decreases the risk of AF occurrence in RA patients [159]. MTX has the potential to prevent the formation and progression of abdominal aorta aneurysm (AAA). However, initial evidence comes from preclinical studies and further research is required to evaluate the effectiveness of MTX in AAA management [160]. 

### 3.3. Cyclosporine A (CsA)

Cyclosporine A (CsA) is an immunosuppressive drug belonging to the calcineurin inhibitors class. CsA is used in RA, nephrotic syndrome, psoriasis, graft versus host disease, and transplantation to prevent graft rejection. CsA immunosuppressive activity is associated with the inhibition of cytokines synthesis, including IL-2 and IL-4, resulting in impairment of activation, differentiation, and maturation of T lymphocytes [161]. Implementation of CsA in transplantation significantly improved the survival of grafts and patients. On the other side, due to its toxicity on multiple vital organs, its clinical usage is limited and requires careful monitoring. A recent meta-analysis of five randomized controlled trials (RCT) showed that CsA treatment after kidney transplantation is associated with a higher risk of hyperlipidemia and hypertension and a lower risk of DM [162]. Correspondingly, a recently published meta-analysis of 19 atopic dermatitis studies indicates the association between CsA and hypertension [163]. The diabetogenic potential is one of the major adverse events of CsA. However, compared to other immunosuppressants used in post-transplantation management, CsA is associated with the least risk of diabetes [164]. Therefore, changing the tacrolimus on the CsA is recommended in the case of post-transplant diabetes mellitus. Nogueiras-Álvarez et al., in a recently published retrospective study of lung transplant recipients, demonstrated that CsA treatment is associated with an increased risk of de novo arterial hypertension and a lower risk of dyslipidemia compared to other immunosuppressant tacrolimus [165]. In opposition, a recent meta-analysis of 13 studies demonstrates that CsA is associated with a higher risk of dyslipidemia compared to tacrolimus in patients after renal transplantation [166]. According to guidelines, the first line of treatment in CsA-induced dyslipidemia is statins. Ezetimibe as a second-line treatment should be considered in case of statin inefficiency or intolerance [167]. The concomitant use of statins and CsA is associated, due to a common metabolism pathway, with an increased risk of serious adverse events such as rhabdomyolysis. CsA-induced changes in lipid profile may enhance atherosclerosis development. However, extensive cohort studies evaluating the association between CsA and premature atherosclerosis are lacking. Su et al., in a preclinical murine model study, showed that CsA promotes atherosclerosis through suppression of microRNA-204 transcription [168]. Conversely, in the cross-sectional study conducted on a small cohort (88 patients) of pediatric liver transplant recipients, it has been demonstrated that patients on CsA treatment had lower carotid intima–media thickness compared to the tacrolimus group [169]. 

The effects of glucocorticoids, methotrexate, and cyclosporin A on the cardiovascular system are shown in Table 2.

### 3.4. Anthracyclines

In the context of drug-induced immunodeficiency, it is crucial to consider the impact of antineoplastic drugs utilized in cancer treatment. Anthracyclines are one of the most effective and commonly used antineoplastic drugs. They are used against acute leukemias, lymphomas, sarcomas, and breast, lung, bladder, and cervical cancers. The cytotoxic effect of anthracyclines is associated with the inhibition of nucleic acid synthesis through several mechanisms [170]. In contrast to the aforementioned agents, anthracyclines are not classified as immunosuppressive drugs. However, they do induce secondary immunodeficiency due to the induction of myelosuppression. Anthracyclines-induced myelosuppression is associated with the inhibition of nucleic acid synthesis in hematopoietic stem cells in the bone marrow. It is possible for neutropenia and leukopenia to develop in up to 70% of patients who receive anthracyclines. A reduction in the number of white blood cells (WBCs) compromises the host’s immune system, increasing the risk of severe infections, sepsis, and mortality [171]. Neutropenia typically emerges 10 to 14 days following the administration of the agent and persists for up to four weeks following the discontinuation of anthracyclines [172]. One of the main limitations of anthracycline use is its cardiotoxicity manifested as arrhythmias, cardiomyopathy, or heart failure [173]. Anthracyclines-induced cardiomyopathy is strongly dose-dependent and occurs in 5–20% of patients. However, cardiac systolic dysfunction may develop even after a low-dose regimen of anthracyclines which suggests that these agents are cardiotoxic at any dosage. There are a few potential mechanisms underlying the cardiotoxic effect of anthracyclines. Anthracyclines induce apoptosis of cardiomyocytes through the promotion of oxidative stress, cardiolipin sequestering, calcium overload, or mitochondrial dysfunction. Anthracyclines can also cause DNA damage within cardiomyocytes [174]. Treatment with anthracycline is associated with significant cardiovascular risk, where such patients require complex cardiological care. The recent consensus of the International Cardio-oncology Society (IC-OS) emphasizes the role of routine left ventricular ejection fraction (LVEF) monitoring with an echocardiogram during anthracycline therapy. Assessment should be repeated after achieving a higher cumulative dose of anthracycline. Notably, a fall in LVEF <50% even in asymptomatic patients may cause discontinuation of anthracycline therapy and decrease patient prognosis. Monitoring changes in biomarkers such as B-type natriuretic peptide (BNP), N-terminal pro-BNP (NT-proBNP), and cardiac troponins may help in the early detection of anthracycline-induced cardiotoxicity. Elevation of natriuretic peptides compared to a pre-treatment level may help establish the diagnosis of heart failure. At the same time, the increase of cardiac troponin above the 99th percentile limit strongly suggests cardiac injury. Notable clinical decisions of anthracycline discontinuation should not be based only on the elevation of biomarkers but also on cardiac imaging with an echocardiogram [175]. The cardiotoxic effect of anthracyclines may be decreased through several potential strategies. First, anthracycline administration should be through slow infusion (24–96 h) rather than fast bolus. The use of liposomal anthracyclines is associated with decreased penetration to the heart tissue, together with increased penetration to tumor tissue [170]. Concomitant administration of anthracyclines and dexrazoxane, which is an iron chelator approved in adults and children treated with cumulative doses equivalent to >300 mg/m^2^ of doxorubicin, provides cardiac protection through decreasing the oxidative stress and DNA damage in cardiomyocytes [172]. Carvedilol, a beta blocker used in hypertension and chronic heart failure, may prevent anthracycline cardiotoxicity through the reduction of reactive oxygen species formation, calcium overloading, and apoptosis of cardiomyocytes [174]. Potential cardioprotection may also be achieved through the administration of ω-3 polyunsaturated fatty acids [170].

### 3.5. Cyclophosphamide

Cyclophosphamide (CY) is an alkylating anticancer and immunosuppressive agent. CY is applied in cancer treatment, including ovarian, lung, and breast cancer and hematological malignancies such as acute and chronic leukemias, lymphoma, and multiple myeloma. Due to its immunosuppressive activity, CY is used in organ transplantation and autoimmune diseases such as multiple sclerosis, granulomatosis with polyangiitis, and lupus nephritis [176]. CY inhibits immune response through several mechanisms, including impairment of the phagocytic activity of the macrophages, downregulation of cytokines such as IFN-γ, IL-2, IL-4, and IL-6, and interference with proliferation and differentiation of T and B lymphocytes [177,178]. CY may also cause immunodeficiency through its depressive impact on bone marrow function. CY damages the hematopoietic stem cells, which results in myelosuppression [179]. Apart from the myelotoxic effect of CY, another significant limitation of its use in clinical practice is cardiotoxicity. Cardiotoxicity affects 8–20% of adults at doses >120–150 mg/kg, albeit it may occur even if the dose is less than 120 mg/kg [180]. The cardiotoxic effect of cyclophosphamide is manifested as hemorrhagic myocarditis, cardiomyopathy, heart failure, arrhythmias such as atrial fibrillation, pectoral angina, and acute myocardial infarction [173]. The mechanisms underlying CY cardiotoxicity include, e.g., cardiomyocyte apoptosis, cardiac inflammation, oxidative stress, calcium dysregulation, and mitochondrial damage [181]. The cardiotoxic effect of CY is associated with the total dose of CY, the patient’s age, the type of malignancy, and the patient’s prior history, including cardiovascular diseases [182]. The risk of cardiac toxicity necessitates the monitoring of CY treatment. A recent position statement from the Cardio-Oncology Council of the European Society of Cardiology provides valuable and transparent guidelines for cardiotoxicity screening in cancer patients. The frequency of monitoring is associated with the baseline cardiovascular risk (CVR); however, all patients should have an echocardiogram evaluation and measurement of natriuretic peptides and cardiac troponin at the baseline and 12 months after treatment regardless of the initial CVR. The frequency of monitoring is increased when the patient’s CVR is higher [183]. The early echocardiographic markers of CY cardiotoxicity include diastolic dysfunction, the thickness of the interventricular septum in diastole, increased left ventricular diastolic/systolic diameter, and mitral regurgitation. In the context of echocardiographic imaging in patients with CY-induced hemorrhagic myocarditis, the following findings are observed: hypertrophy, myocardial hyperechogenicity, a decrease in LVEF, and no change in ventricular sizes. Nevertheless, cardiac magnetic resonance imaging (MRI) remains the gold standard for the diagnosis of CY-induced myocarditis. Notably, CY should be discontinued when any clinical, laboratory, or radiographic signs of cardiotoxicity are present [182].

## 4. Malnutrition

Malnutrition is a multifaceted condition characterized by deficiencies or imbalances in essential nutrients. It represents a significant global health challenge with profound implications for immune function, and therefore, it can negatively impact the cardiovascular system. Given the web of interactions within the human body, the symbiotic relationship between nutrition and immunity emerges as a crucial element of physiological well-being. In industrial countries, the most common reason for malnutrition is diseases [184]. The immune system is highly dependent on the nutrients that are delivered with the consumed food. All nutrients have to be delivered in the right amount. Essential nutrients, including vitamins, minerals, and macronutrients (especially proteins), serve as indispensable cofactors in various immune processes, ranging from the differentiation and proliferation of immune cells to the production of cytokines and antibodies. Deficiencies in these vital components undermine the immune system’s ability to mount effective defenses against pathogens, leaving individuals vulnerable to infectious diseases, impaired wound healing, and chronic inflammatory conditions. When this delicate balance is disrupted by malnutrition, a cascade of immunological consequences occurs, precipitating a state of secondary immunodeficiency [185]. 

Malnutrition correlates with compromised gut–barrier integrity, decreased secretion of protective substances, and diminished plasma complement concentration. The lymphatic system, especially the thymus, experiences atrophy, leading to reduced delayed-type hypersensitivity responses. Severely malnourished children exhibit diminished antibody production post-vaccination, while those with moderate malnutrition maintain normal levels. Immune response patterns often shift towards a Th2 bias. However, certain immune parameters remain unaffected or elevated, including leukocyte and lymphocyte counts, along with high levels of immunoglobulins, particularly immunoglobulin A. Early-life undernutrition increases the risk of obesity in later life. Malnutrition significantly impacts gut function, leading to conditions like environmental enteric dysfunction (EED), characterized by impaired gut structure and function. EED is often caused by repeated exposure to enteric pathogens in unsanitary conditions. Mechanisms of EED include altered nutrient-sensing pathways, tissue damage releasing immune-activating molecules, and compromised gut barrier function, leading to systemic microbial leakage. Immune dysregulation in EED contributes to poor linear growth and stunting, mediated by pro-inflammatory mediators like IL-6 and dysregulated growth hormone signaling. Murine models of EED show reduced gut integrity, altered microbiota, and increased susceptibility to infections, highlighting the role of infection-driven immune dysfunction in growth failure. Additionally, chronic immune activation, as seen in EED, can independently lead to wasting and increased susceptibility to infections, further exacerbating the effects of malnutrition [185,186,187].

It is generally acknowledged that”unde’nutrition and malnutrition have a detrimental impact on the immune system and cardiovascular health. However, there is a need for further research to elucidate the full extent of this impact. The currently available data provides insights into the roles of proteins, polyunsaturated fatty acids, vitamin B6, vitamin D, and magnesium in maintaining cardiovascular well-being. Nevertheless, further comprehensive research in this domain is imperative to deepen the understanding and uncover novel therapeutic avenues. Unfortunately, malnutrition currently occurs not only in populations such as children, the elderly, and those affected by poverty or food insecurity, but due to the practices of the food industry, also in people with excessive and normal body weight. We are dealing with a deficiency of non-nutritive bioactive compounds, including vitamins and minerals [188,189].

### 4.1. Proteins

Protein is a crucial component of the diet, playing a fundamental role in supporting the body’s immune system. Numerous studies have elucidated the significant impact of protein deficiency on immune function, highlighting the importance of adequate protein intake for maintaining optimal immune responses. A study on guinea pigs emphasizes the essential role of dietary protein in supporting the production of antibodies, which are vital for recognizing and neutralizing pathogens in the body [190]. Inadequate protein intake can impair antibody synthesis, compromising the body’s ability to mount effective immune responses against infections. Moreover, protein deficiency can lead to alterations in the distribution and function of immune cells. T cells, B cells, and natural killer (NK) cells, critical components of the immune system, require adequate protein for their development and activation. A deficiency in dietary protein can reduce the number and activity of these immune cells, weakening the body’s defense mechanisms [191]. The paucity of contemporary research utilizing sophisticated diagnostic techniques hampers the precise determination of the impact of protein-energy malnutrition on immune system function.

### 4.2. Polyunsaturated Fatty Acids

The health benefits of omega-3 PUFA have been extensively researched. The balance between eicosapentaenoic acid (EPA) or Docosahexaenoic acid (DHA) and arachidonic acid (AA) in the human body appears to be substantial for regulating the production of mediators and, subsequently, vascular function. Indeed, the serum EPA to AA ratio (EPA/AA) has been found to be a promising biomarker for CVD risk [192]. While the exact mechanisms underlying the cardiovascular benefits of omega-3 intake remain unclear, recent studies offer novel perspectives. Some of these benefits may arise from metabolites with potent anti-inflammatory properties. For instance, metabolites derived from EPA, such as E-series resolving, actively inhibit leukocyte migration to inflammatory sites, enhance the removal of inflammatory cells, and suppress cytokine release [193]. Moreover, omega-3 fatty acids have been demonstrated to decrease pro-inflammatory cytokines such as interleukin-6 and tumor necrosis factor-α and inhibit the activation of the IκB kinase and nuclear factor-κB, as well as several other transcription factors that inhibit reactive oxygen species [194].

### 4.3. Vitamin B6

B6 supports both innate and acquired immunity. Possible mechanisms include inhibition of inflammation by stopping the cytokine storm and oxidative stress. It also plays a part in the regulation of Ca^2+^ levels and increasing carnosine levels [195]. Carnosine is a cardioprotector, with its physiological role being intramuscular pH buffering, the management of intramuscular calcium levels, enhanced sensitivity of the contractile apparatus to calcium, improvements in histopathological and hemodynamic parameters, the reduction of reactive oxygen species (ROS), metal ion chelation, and protection against lipid peroxidation [196]. Vitamin B6 might regulate cellular calcium influx via voltage-gated ATP-dependent purinergic receptors, suggesting its involvement in hypertension and cardiac function regulation [195,197]. Imidazole dipeptides are believed to contribute to cardiac protection. Pyridoxal phosphate (PLP), which is an active form of B6 vitamin, governs the balance of cardio-protective substances such as imidazole dipeptides (like carnosine, homocarnosine, and anserine) known for their antioxidant and anti-inflammatory properties. Kumurgsee et al.’s review underscores vitamin B6’s contribution to cardiac protection by affecting histamine, GABA, and imidazole dipeptides, as well as by inhibiting the P2 × 7R-NLRP3 inflammasome. Furthermore, the modulation of anserine, carnosine, histamine, GABA, and the P2X7R–NLRP3 inflammasome appears to mitigate inflammation and oxidative stress [197]. Additionally, PLP plays an important part in transaminase reactions. Almost all PLP-dependent enzymes are associated with biochemical pathways involving amino acids pathways. Those enzymes act on amino acids, mostly including transamination, decarboxylation, and racemization [198]. For example, in the alanine aminotransferase (ALT) reaction, PLP helps transfer an amino group from alanine to alpha-ketoglutarate, forming pyruvate and glutamate, which serve as precursors of the synthesis of some amino acids such as alanine, serine, cysteine, and glycine [199]. Those amino acids are essential for the proliferation and differentiation of immune cells, particularly lymphocytes and T cells. Glycine is crucial in suppressing the production of pro-inflammatory cytokines like TNF-alfa, IL-6, and IL-1β [200]. Vitamin B6, through its role in transaminase reactions, also participates in the metabolism of homocysteine. This amino acid is associated with an increased risk of cardiovascular diseases when present in elevated levels [201]. Homocysteine is proven to be an independent risk factor for atherosclerosis. It also has adverse effects on vascular endothelium and smooth muscle cells with resultant alterations in subclinical arterial structure [202]. Proper conversion of homocysteine to cysteine requires PLP-dependent enzymes [201]. As shown, vitamin B6 plays an integral part in keeping cardiovascular health through various mechanisms.

### 4.4. Vitamin D

Vitamin D is essential for overall health and can be obtained from sunlight exposure as well as dietary sources such as fatty fish, fortified dairy products, and egg yolks. Although traditionally associated with bone health, the demonstration of the presence of a specific receptor for vitamin D in the heart suggested a direct role of vitamin D in maintaining cardiovascular function [198]. Deficiency in vitamin D can promote the sustained activation of the renin–angiotensin–aldosterone system (a marked increase in renin expression and angiotensin II production has been observed in mice and humans with inactivated vitamin D receptors), increasing angiotensin with the consequences of arterial hardening, endothelial dysfunction and the development of hypertension. Numerous animal models and observational studies in humans strongly support the hypothesis that vitamin D deficiency contributes to high blood pressure. However, it should be noted that the antihypertensive effect of vitamin D was not observed in some trials, which could be due to a suboptimal study design, the knowledge that a deficiency of vitamin D could promote the sustained activation of RAAS (renin–angiotensin–aldosterone system), increasing angiotensin, arterial hardening, and endothelial dysfunction, could support the hypothesis that vitamin D deficiency contributes to the development of hypertension [199]. Both the vitamin D receptor and 1α-hydroxylase are found in vascular tissues such as endothelial cells, vascular smooth muscle cells (VSMCs), and cardiomyocytes. In the vascular wall, the active form of vitamin D, 1,25-dihydroxyvitamin D (1,25(OH)2D), exerts various beneficial effects, including reducing thrombogenicity, lowering vasoconstrictors, inhibiting oxidative stress and atherogenesis, enhancing endothelial repair, reducing foam cell formation, and promoting vascular relaxation and dilation. However, 1,25(OH)2D can also induce VSMCs to transform into osteoblast-like cells, potentially leading to vascular calcification. In cardiomyocytes, 1,25(OH)2D regulates intracellular calcium metabolism through both genomic and non-genomic mechanisms. Evidence suggests that the synthesis of 1,25(OH)2D in the heart and vasculature is regulated by parathyroid hormone (PTH) and fibroblast growth factor-23 (FGF-23). Additionally, FGF-23 induces hypertrophic growth in cardiomyocytes. While the exact mechanisms of vitamin D signaling in cardiomyocytes and the vascular wall remain incompletely understood, available data indicate that 1,25(OH)2D plays a crucial role in maintaining proper cardiac and vascular function. Besides VDR activation, various cardiovascular effects of vitamin D may also involve parathyroid hormone (PTH) [200]. In conclusion, the worsening worldwide trend toward an insufficiency of vitamin D and the growing knowledge of its cardiovascular actions have sparked increasing interest in vitamin D in the prevention and treatment of major cardiovascular diseases [201]. Vitamin D also plays an important part in immune response. Calcitriol enhances the antimicrobial functions of macrophages and monocytes by activating VDR-RXR signaling. This process leads to the production of cathelicidins, which attach to microbial membranes to eliminate bacteria and fungi. Cathelicidin also plays a role in directly targeting various respiratory viruses by disrupting their envelopes. This mechanism is particularly significant in conditions involving granulomatous inflammation, such as tuberculosis (TB), lymphomas, and sarcoidosis. 1,25-(OH)2D3 exerts its effect via direct binding on both VDR on the dendritic cell (CD) and the T lymphocytes [202,203]. 1,25-(OH)2D3 is also responsible for anti-inflammatory activity on macrophages, increases IL-10, and it causes a decrease in pro-inflammatory stimuli such as IL-6, TNF-alfa, receptor activator nuclear factor kappa-B ligand (RANKL), and cyclo-oxygenase-2 (COX-2). This downregulation happens because of the upregulation of mitogen-activated protein kinase (MAPK) phosphatase (MKP)-1 by 1,25-(OH)2D3 and subsequent inhibition of LPS-induced p38 activation [204]. Therefore, vitamin D contributes to cardiovascular health by reducing inflammation and protecting vessels from remodeling.

### 4.5. Magnesium

Magnesium is an essential nutrient for cardiovascular health, acting to regulate vascular smooth muscle, cardiac conduction, vascular endothelial cell functioning, and thrombosis. Hypomagnesaemia and low dietary magnesium intake increase the likelihood of developing coronary artery disease (CAD). Hypomagnesaemia has been associated with hypertension, which can lead to congestive heart failure (HF) or CAD [202]. Magnesium is involved in the homeostasis of blood pressure. It does not have a direct influence on biochemical mechanisms of contraction, but studies have shown that Mg controls vascular smooth muscle contraction [203,204,205]. Magnesium acts as a weak physiologic Ca channel blocker, modulating Ca-channel activity in heart cells [205]. Mg deficiency stimulates the synthesis of aldosterone mediated by angiotensin II, along with increased production of thromboxane and vasoconstrictor prostaglandins. Additionally, magnesium exerts a beneficial effect on vascular endothelium by modulating the release of nitric oxide, prostacyclin, and endothelin-1 [206,207]. The evidence for a beneficial effect of magnesium on hypertension risk emphasizes the importance of broadly encouraging the consumption of foods, such as vegetables, nuts, whole cereals, and legumes, optimal dietary sources of magnesium, and avoiding processed foods, which are very poor in magnesium and lack other fundamental nutrients as well, in order to prevent hypertension [207]. Magnesium is also known for its role in innate immune system and adaptive immunity. In the innate immune system, it effects the acute phase response and the function of macrophages. In monocytes, magnesium has an immunomodulatory function influencing a reduction in cytokine production after toll-like receptor (TLR) stimulation [208]. In terms of adaptive immunity, magnesium has a major influence on the development, differentiation, and proliferation of lymphocytes [209]. The Mg^2+^ transporter TRPM7 seems to play a special role in the development of T cells. Chronic Mg deficiency leads to enhanced baseline inflammation driven by elevated cytokines like IL-6 and C-reactive protein (CRP) and therefore plays a central role in atherosclerosis [210]. A recent review and meta-analysis showed an inverse association between serum Mg^2+^ levels and the incidence of CVD [206].

## 5. SIDs Diagnostics

Diagnostics for SID should primarily include a well-collected medical history. It should also include information on the patient’s age to exclude immaturity of the immune system associated with childhood or old age, nutritional status, addictions, and the ability to cope with stress. A simplified diagnostic scheme in this area is presented in Table 3.

Laboratory diagnostics should include a complete blood count with white blood cell differential, total protein, albumin, folate, vitamin B12, ferritin, vitamin D3, calcium, uric acid, and lipid profile [208]. When the patient has inflammation and it is anticipated that ferritin (an acute phase protein) may be elevated, a transferrin saturation test (TSAT) is recommended, which requires iron concentration and total iron binding capacity. If TSAT is less than 15% in men and 12% in women, iron deficiency can be diagnosed and supplementation is necessary [209]. 

Remember that currently most vitamin B12 deficiencies result from dietary deficiencies, which is why it is worth trying oral supplementation and re-testing the concentration of this vitamin after a few days of supplementation, rather than condemning the patient to lifelong intramuscular supplementation.

If possible, it is worth performing a proteinogram or determining the concentrations of the main immunoglobulin classes, i.e., IgA, IgG, and IgM. The result of the γ-globulin fraction determined in the proteinogram can tell us a lot—a concentration within the lower limit of the norm or reduced may indicate humoral immunity disorders, because it is in this fraction that all antibodies are found in the highest concentration.

Due to the increased risk of infection, diagnostics should be implemented early and inflammatory parameters should be determined (acute phase protein—CRP, or procalcitonin). In selected patients, microbiological diagnostics is necessary.

In selected cases, diagnostics should be supplemented with imaging tests (X-ray, ultrasound, computed tomography, or magnetic resonance imaging).

## 6. Therapeutic Management of SIDs

Therapeutic treatment in SIDs is multidirectional. It should primarily consist in eliminating or limiting the negative effects of these deficiencies on the body. Depending on the cause of SID, treatment includes optimal treatment of the chronic disease, use of drugs with the lowest immunosuppressive and cardiotoxic potential, and optimal nutrition.

In selected cases, it is advisable to administer additional vaccinations, including against pneumococci, the influenza virus, and SARS-CoV-2. This is particularly relevant for patients with heart defects and chronic cardiovascular diseases (e.g., hypertension, chronic heart failure, coronary artery disease, and arrhythmias). Depending on the patient’s health condition and risk factors, the doctor may also recommend other vaccinations, such as vaccination against hepatitis B. This applies especially to heart disease patients who are treated in healthcare facilities or have other risk factors. It is also recommended to receive booster vaccinations against tetanus and diphtheria [210,211].

In cases of severe infections or when antibody production is significantly weakened, patients may be given immunoglobulins intravenously (IVIG) or subcutaneously (SCIG). This therapy provides IgG antibodies, which help combat infections. Such treatment is most commonly used in patients with certain hematologic proliferative diseases. It is important to remember that the immunoglobulin preparation is derived from the serum of approximately 1000 donors, which should be taken into account during diagnostics involving antibody testing [212]. In selected situations, cytokines, such as interferons or growth factors, may be used to stimulate the production and activity of immune system cells, for example, in cases of accompanying neutropenia [213]. In patients at high risk of infection, such as during chemotherapy, prophylactic antibiotics are often used to prevent the development of infections [214,215].

Patients with secondary immunodeficiencies require regular monitoring of their health status, more often than in immunocompetent individuals.

## 7. Conclusions

Chronic diseases such as diabetes, autoimmune disorders (e.g., lupus), kidney disease, or chronic heart failure can lead to chronic inflammation and immune system dysfunction. This results in accelerated atherosclerosis, endothelial damage, and an increased risk of heart attacks and strokes. Chronic or severe infections can lead to vascular inflammation and endothelial damage, increasing the risk of atherosclerosis and thrombosis. They can also lead to complications such as myocarditis and chronic heart failure. Certain immunosuppressive drugs used to treat autoimmune diseases, organ transplants, or cancer can cause metabolic disturbances like dyslipidemia, hypertension, and insulin resistance. These medications (e.g., corticosteroids, calcineurin inhibitors) can increase the risk of developing cardiovascular diseases, including atherosclerosis and hypertension. Severe trauma and surgeries, especially involving major organs or requiring prolonged hospitalization, can trigger inflammation and coagulation system activation. Prolonged immobilization, post-traumatic stress, and metabolic changes promote the formation of clots and emboli, increasing the risk of heart attacks and strokes. Some genetic disorders associated with immune deficiencies may also predispose individuals to cardiovascular diseases. For instance, Marfan syndrome, which often coexists with heart defects, increases the risk of aneurysms and vascular abnormalities. Exposure to radiation (e.g., radiation therapy for cancer treatment) and certain chemical toxins can lead to vascular damage and an increased risk of atherosclerosis. Radiation therapy to the chest area can cause pericarditis, coronary artery atherosclerosis, and heart failure due to direct damage to heart tissue. Each of these factors weakens the immune system, and the accompanying inflammation directly contributes to vascular and heart damage, increasing the risk of serious cardiovascular diseases.

Cardiovascular diseases have a profound impact on the entire body. Conversely, dis-eases of various organs and systems can affect the onset or course of cardiovascular diseases. Consequently, it is imperative to consider primary and secondary immuno-deficiencies in the care and treatment of cardiac patients. Although secondary immunodeficiencies are more common than primary ones, they can coexist. Therefore, when evaluating a cardiac patient, it is important to assess not only age, gender, body weight, serum lipid levels, or smoking status, but also comorbidities, especially those with metabolic, autoimmune, or proliferative disorders, as well as infections. It is of particular importance to consider medications used, as well as nutritional status with an assessment of nutrient deficiencies and non-nutritive bioactive compounds. Only a holistic assessment of the patient, which considers the aforementioned factors, provides the best opportunity for the optimal management of cardiovascular disease. It is evident that this group of patients requires additional education and more frequent examinations and follow-up visits.

## Figures and Tables

**Table 1:** Secondary immunodeficiencies [1,2,3,4,5,6]. SARS-CoV-2—Severe Acute Respiratory Syndrome Coronavirus 2, CMV—*Cytomegalovirus*; EBV—*Epstein–Barr Virus*; HIV—*human immunodeficiency virus*; IL—interleukin; JAK—Janus kinase; TNF—tumor necrosis factor.

Deficiency Category	Selected Detailed Causes
Systemic Diseases:	Metabolic disorders: e.g., diabetes, obesity Autoimmune diseases: e.g., systemic lupus erythematosus, rheumatoid arthritis Proliferative diseases: e.g., leukemia, lymphomas, multiple myeloma, solid organ cancers Gastrointestinal diseases: e.g., inflammatory bowel disease, enteropathies, severe diarrhea, liver diseases such as cirrhosis and hepatitis Kidney diseases: e.g., nephrotic syndrome Protein deficiencies: e.g., starvation, cachexia Increased catabolism: e.g., cancer, hyperthyroidism, sepsis
Infections:	Viral infections: e.g., SARS-CoV-2, CMV, EBV, HIV, parvovirus B19, rubella, measles, herpes, Bacterial infections: e.g., tuberculosis Parasitic infections: e.g., toxoplasmosis (including congenital), malaria
Medications:	Immunomodulatory and immunosuppressive drugs: e.g., glucocorticoids, sulfasalazine, gold salts, penicillamine, mycophenolate mofetil, methotrexate, cyclosporine, cyclophosphamide, hydroxychloroquine, leflunomide, various biological drugs Biologics: e.g., JAK inhibitors (baricitinib, tofacitinib, upadacitinib, ruxolitinib), TNF-a inhibitors (certolizumab, adalimumab, infliximab, golimumab, etarnecept), monoclonal antibodies targeting B cells (rituximab, veltuzumab, ocrelizumab, ofatumumab, binutuzumab, ublituximab, epratuzumab, blinatumomab, alemtuzumab, daratumumab, isatuximab, belimumab), IL-1 inhibitor (anakinra, canakinumab), IL-6 inhibitor (sarlimumab, tocilizumab), cancer immunotherapy (ipilimumab, nivolumab and pembrolizumab, atezolizumab, durvalumab) Cancer chemotherapy: e.g., angiogenesis inhibitors (bevacizumab), antimetabolites (5-fluorouracil), anthracyclines (doxorubicin), anthracendiones (mitoxantrone), glycopeptide antibiotics (bleomycin) Antiepileptic drugs: e.g., carbamazepine, phenytoin, sodium valproate, lamotrigine Antipsychotic drugs: e.g., chlorpromazine Other drugs: e.g., captopril, diclofenac
Trauma and Surgery:	Multiorgan injuries Burns Extensive surgeries Splenectomy
Hereditary Diseases:	Metabolic disorders: e.g., trans-cobalamin II deficiency with hypogammaglobulinemia Chromosomal aberrations: e.g., monosomy 22, trisomy 8 and 21, chromosome 18q syndrome
Radiation and Toxins:	Exposure to ionizing radiation: e.g., radiotherapy Toxic chemicals: e.g., benzene, solvents
Other Causes:	Pregnancy Plasma exchange transfusion Absence or dysfunction of the spleen Stress

**Table 2:** The effects of glucocorticoids, methotrexate, and cyclosporine A on the cardiovascular system. CVD—cardiovascular disease, CVE—cardiovascular events, RA—rheumatoid arthritis, SLE—systemic lupus erythematosus, TG—triglycerides, TC—total cholesterol, LDL-C: low-density lipoprotein cholesterol, ApoB—Apolipoprotein B, HDL-C: high-density lipoprotein cholesterol, ApoA1—Apolipoprotein A1, pAS—premature atherosclerosis, CRP—C-reactive protein, RR—relative risk, CI—confidence interval, DM2—type 2 diabetes mellitus, AI—atherosclerosis index, AF—atrial fibrillation.

Drug	Disease	Effect	Study Design	Ref.
Glucocorticoids (GCs)	Immune-mediated inflammatory diseases	The increased cumulative risk of all-cause CVD; GCs are associated with dose-dependent risk of atherosclerotic diseases, heart failure, atrial fibrillation, and abdominal aortic aneurysms.	Retrospective, population-based cohort study	[136]
	Chronic inflammatory diseases	Cumulative dose of GCs (but not daily dose) is associated with an increased incidence of hypertension; Giant cell arteritis is associated with the highest cumulative dose of GCs; GCs may prevent hypertension development in polymyalgia rheumatica	Retrospective, population-based record-linkage cohort study	[140]
	RA	Daily dose ≥7.5 mg of prednisolone equivalent dose significantly increases the risk of hypertension;	Retrospective, cohort study	[141]
	SLE	GCs use in SLE patients is associated with an increased risk of diabetes	Meta-analysis	[143]
	SLE	≥30 mg/day prednisone significantly increases serum concentration of TG, TC, LDL-C, and ApoB and decreases HDL-C and ApoA1.	Retrospective, single-center study	[147]
	SLE	Duration of GCs therapy together with other factors including hypertension, antiphospholipid syndrome, azathioprine use, and age of patient are independent indicators for premature atherosclerosis (pAS). The authors proposed a scoring system that allows for the prediction of pAS incidence in SLE patients	Cross-sectional study	[148]
	RA	GCs use combined with age, male gender, smoking, hyperlipidemia, hypertension, diabetes, and CRP concentration are associated with increased risk of coronary artery disease	Meta-analysis	[149]
Methotrexate (MTX)	RA	MTX use is independently associated with a significant reduction in mortality from CVD in RA	Meta-analysis	[152]
	RA	MTX use is associated with a significantly decreased risk of CVE (RR = 0.798, 95% CI 0.726–0.876, *p* < 0.001)	Meta-analysis	[153]
	RA	MTX and regular exercises are associated with a decreased risk of hypertension in RA patients	Systematic review	[152]
	RA	MTX decreases the risk of DM2 development in RA patients (RR 0.48, 95% CI 0.16, 1.43)	Meta-analysis	[155]
	Diet-induced dyslipidemia	MTX significantly decreases ongoing inflammation and oxidative stress; MTX significantly decreases the concentration of TC, LDL-C, TG, and AI; MTX treatment significantly decreases the thickness of media and intima in the murine aorta.	Preclinical study (murine model)	[156]
	RA	≥20 mg/week of MTX significantly lower intima–media thickness in carotid and femoral arteries; MTX treatment correlates negatively with the presence of atherosclerotic plaques in carotid and femoral arteries	N/A	[157]
	RA	The use of MTX decreased the risk of incident AF. In patients ≥50 years MTX decreased the occurrence of AF in males	Case-control study	[159]
Cyclosporine A (CsA)	Kidney transplantation	CsA treatment is associated with a higher incidence of hyperlipidemia and hypertension but a lower rate of diabetes compared to tacrolimus treatment.	Meta-analysis	[162]
	Atopic dermatitis	Systemic CsA is associated with an increased incidence of hypertension	Meta-analysis	[163]
	Post-transplant diabetes mellitus (PTDM)	Cyclosporine treatment in kidney transplant recipients is significantly less diabetogenic than tacrolimus and sirolimus	Meta-analysis	[164]
	Lung transplantation	CsA is associated with a higher rates of developing hypertension and renal function impairment development and a lower rates of hyperlipidemia compared to tacrolimus	Retrospective, observational study	[165]
	Kidney transplantation	CsA is associated with a lower risk of new-onset diabetes and a higher risk of dyslipidemia compared to tacrolimus; No difference in patient survival between CsA and tacrolimus	Meta-analysis	[166]
	Liver transplantation	Carotid intima–media thickness was significantly lower in CsA group compared to the tacrolimus group	Cross-sectional study	[169]

**Table 3:** Simplified scheme of the SIDs diagnostics.

**Last and first name:**		
**Cardiological disease:**		
**Parameter**	**Yes**	**No**
**Age**		
<16 years of age >65 years of age	□ □	□ □
**Chronic diseases**		
Type 2 diabetes mellitus Chronic kidney disease Rheumatological disease Inflammatory bowel disease Other autoimmune disease Neoplasms Chronic infections Other……………………………	□ □ □ □ □ □ □ □	□ □ □ □ □ □ □ □
**Medicaments**		
Immunosuppressive medications Cardiotoxic medications Other……………………………	□ □ □	□ □ □
**Nutritional status**		
Malnutrition Normal weight Overweight Obesity	□ □ □ □	□ □ □ □
**Deficiency of non-nutritive bioactive compounds** (e.g., extensive information about the diet used)		
Suspected deficiency	□	□
**Addiction**		
Alcohol Drugs Medicines Other	□ □ □ □	□ □ □ □
**Stress coping assessment**		
Very good Satisfactory Unsatisfactory	□ □ □	□ □ □
**Other information**		
…………………. e.g., hereditary diseases (including primary immunodeficiencies)	□	□
**Conclusions:**		

## Data Availability

No new data were created or analyzed in this study. Data sharing is not applicable to this article.
